# Robust Superpixel Segmentation for Hyperspectral-Image Restoration

**DOI:** 10.3390/e25020260

**Published:** 2023-01-31

**Authors:** Ya-Ru Fan

**Affiliations:** 1 School of Mathematics, Southwest Minzu University, Chengdu 610041, China; yarufanfan@163.com; 2 School of Mathematical Sciences, University of Electronic Science and Technology of China, Chengdu 611731, China

**Keywords:** hyperspectral-image restoration, robust superpixel segmentation, weighted nuclear norm, principal component

## Abstract

Hyperspectral-image (HSI) restoration plays an essential role in remote sensing image processing. Recently, superpixel segmentation-based the low-rank regularized methods for HSI restoration have shown outstanding performance. However, most of them simply segment the HSI according to its first principal component, which is suboptimal. In this paper, integrating the superpixel segmentation with principal component analysis, we propose a robust superpixel segmentation strategy to better divide the HSI, which can further enhance the low-rank attribute of the HSI. To better employ the low-rank attribute, the weighted nuclear norm by three types of weighting is proposed to efficiently remove the mixed noise in degraded HSI. Experiments conducted on simulated and real HSI data verify the performance of the proposed method for HSI restoration.

## 1. Introduction

Hyperspectral images (HSIs) reflect the spectrum image of a real scene in hundreds of continuous wavebands. Thus, HSIs contain abundant spectral information and have been applied in various fields, such as terrain exploration, environmental monitoring, and military surveillance. Unfortunately, due to the limitation of devices and environmental factors, HSIs are often corrupted by various kinds of noise during data acquisition and transmission. Therefore, HSI restoration is a significant pretreatment, and affects the performance of the subsequent applications, including classification [1,2,3], unmixing [4,5,6], object detection [7,8], and super-resolution [9,10].

In the recent decade, based on the spatial-spectral prior knowledge of HSI, numerous HSI restoration methods have been proposed [11,12,13,14,15]. For instance, paper [11] presented a spectral-spatial adaptive total variation denoising strategy. Article [12] displayed a spectral-spatial structured sparse representation in the HSI restoration model. The group sparse nonnegative matrix factorization (GSNMF) method for HSI restoration was proposed in [13]. With the group sparse regularization term, spectral signatures shared a common set of bases for HSI reconstruction in [14].

The strong correlation of adjacent bands and adjacent pixels ensures that the clean HSI has an underlying low-rank character. Inspired by this prior knowledge, many low-rank matrix or tensor-based methods have been proposed for HSI denoising. For instance, the low-rank matrix recovery (LRMR) approach [16] reformulated the cubic HSI data into a low-rank matrix and adopted the GoDec algorithm to solve the restored problem. The weighted nuclear norm and total variation (WNNTV)-based HSI restoration approach [17] was proposed to remove the hybrid noises of sparse noise and Gaussian noise. The multi-channel weighted nuclear norm minimization (MCWNNM) algorithm [18] considered the low-rank prior both across the spectra and in the spatial domain to well restore the HSI. It can be seen that the weighted nuclear norm for characterizing low-rank prior knowledge of the HSI is a powerful tool on mixed-noise removal.

By retaining the cubic structure of HSI, the rank-1 tensor decomposition [19] and patch-based low-rank tensor decomposition [20] were proposed for HSI noise reduction. To jointly consider the sparse prior knowledge of the HSI, in [21], the total variation regularization was introduced into the low-rank tensor decomposition model. The weighted group sparsity regularized low-rank tensor decomposition (LRTDGS) method was proposed in [22], where the spatial-spectral correlation from three directions of HSI was depicted by low-rank tucker decomposition. Moreover, tensor-completion-based HSI recovery strategies have occurred by exploiting the low-rankness on the pixel level of the HSI [23,24,25]. Both of them achieved the good denoising results, but the tensor rank problem is still an open issue.

According to adjacent position and similar texture feature, pixels are grouped into a homogeneous region (superpixel) of adaptive shape, which can better adhere to the image boundaries than square patches. Recently, superpixel segmentation-based methods for HSI denoising have sprung up. In [26], superpixel segmentation and low-rank representation (SSLRR) for HSI denoising was presented, which employed entropy rate superpixel segmentation (ERS) [27] to divide the HSI based on its first principal component (PC) and formulated the low-rank property of superpixel as the standard nuclear norm in the denoising model. Due to the first PC lacking some detail and boundary information of the HSI, the superpixel segmentation result of the HSI may be suboptimal. Article [28] proposed to divide the HSI via ERS based on a smooth band. The smooth band contained the most significant information of the HSI and could suppress a lot additive noise. It is suggested that the good superpixel segmentation result could improve the HSI restoration.

**Figure 1:**
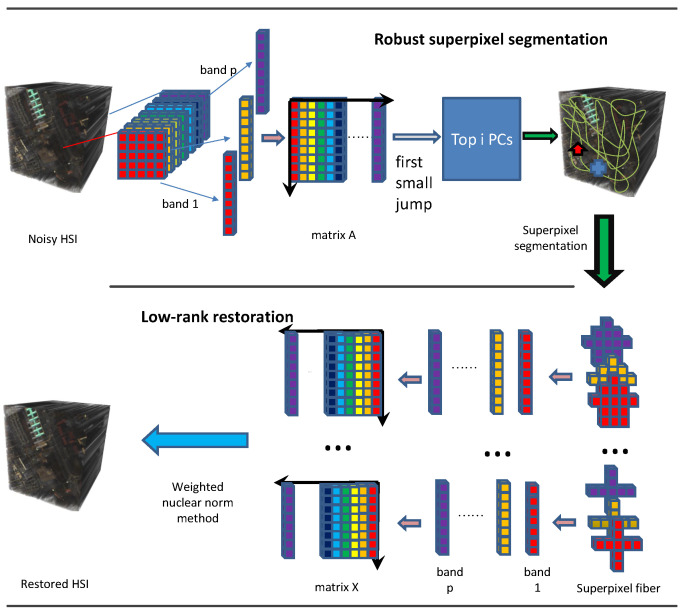
Flowchart of the proposed HSI restoration method.

To obtain a good superpixel segmentation result, in this paper, we combine the superpixel segmentation with principal component analysis to propose a robust superpixel segmentation strategy. The strategy aims to choose some PCs that can explain significant information, including the details and boundaries of the HSI. Based on the selected PCs such as an accurate segmentation map, the HSI can be better divided by superpixel segmentation. The divided superpixel fibers hold low-rank property due to the high correlation within adjacent pixels and bands. The weighted nuclear norm by three types of weighting is adopted to characterize the low-rank property. The flowchart of the proposed approach is displayed in Figure 1.

The contributions of the proposed work are summarized as follows:

- Combining the superpixel segmentation with principal component analysis, we propose a robust superpixel segmentation strategy to better divide the HSI. A “first small jump” scheme in the robust superpixel segmentation is devised to select the PCs. The selected PCs include most of the important information of the HSI. The superpixel segmentation result based on the selected PCs is relatively accurate.
- The weighted nuclear norm is used to characterize the low-rank attribute of superpixel fibers. In particular, we summarize the weighted nuclear norm by three types of weighting in the HSI restoration model.
- The alternating direction method (ADM) is used to solve the weighted-nuclear-norm-based HSI restoration model, and the corresponding iterative optimization process is derived. We adopt three operators in one frame to solve the subproblem with the weighted nuclear norm. The experimental results obtained by different operators are displayed.

The remainder of this paper is organized as follows. The robust superpixel segmentation strategy and the proposed model for HSI restoration are introduced in Section 2. Section 3 presents the algorithm for solving the proposed model. Extensive experiment results are reported in Section 4. Section 5 shows the discussion of the proposed approach. Finally, Section 6 concludes this paper.

## 2. Materials and Methods

### 2.1. Robust Superpixel Segmentation

**Figure 2:**
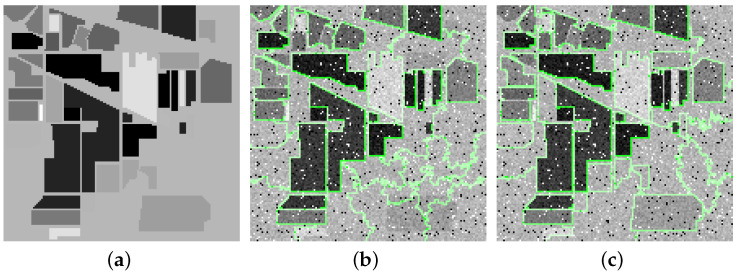
Superpixel segmentation results on noisy Indian Pines. (**a**) Clean band, (**b**) segmentation result based on first PC, and (**c**) segmentation result based on top six PCs.

Superpixel fiber is a cluster of similar pixels along the spectral dimension. Compared with the square fiber in cubic HSI, superpixel fibers with an adaptive shape can better hold the edges and details of the HSI, which guarantees that more spectral-spatial features are extracted. In other words, superpixel segmentation can further enhance the low-rank properties of the HSI. Many HSI denoising methods based on superpixel segmentation simply segment the HSI according to its first PC [26,29,30]. It is suboptimal since the first PC lacks some details and edges of the HSI. In Figure 2, taking a noisy HSI (Indian Pines) as an example, we, respectively, present the ERS results based on the first PC and top six PCs. Obviously, the segmentation result based on the top six PCs is more accurate than the one based on the first PC.

Since each PC of the degraded HSI has a significant amount of noise, selecting too many PCs is not good. A vital problem is how many PCs are appropriate for the good HSI segmentation result. Before solving the problem, we present the variance contribution rate of the *k*th PC of the HSI $y\in R^{m\times n\times p}$ as follows:

(1) $${\hat{r}}_{k}=\frac{\epsilon_{k}}{\sum_{i=1}^{p}\epsilon_{i}},k=1,2,\ldots ,p,$$

where $\epsilon_{i}$ denotes the *i*th eigenvalue of the covariance matrix of matrix $A\in R^{mn\times p}$, in which each column is the vectorized band of *y* (see Figure 1).

Then cumulative variance contribution rate (CVCR) of top *k* PCs is defined as

(2) $$r_{k}=\frac{\sum_{i=1}^{k}\epsilon_{i}}{\sum_{i=1}^{p}\epsilon_{i}},k=1,2,\ldots ,p.$$

Obviously, both the values of ${\hat{r}}_{k}$ and $r_{k}$ are between 0 and 1. With the increasing *k*, ${\hat{r}}_{k}$ becomes smaller, but $r_{k}$ becomes larger and more closed 1. Larger $r_{k}$ indicates that the top *k* PCs hold more information of the HSI.

**Figure 3:**
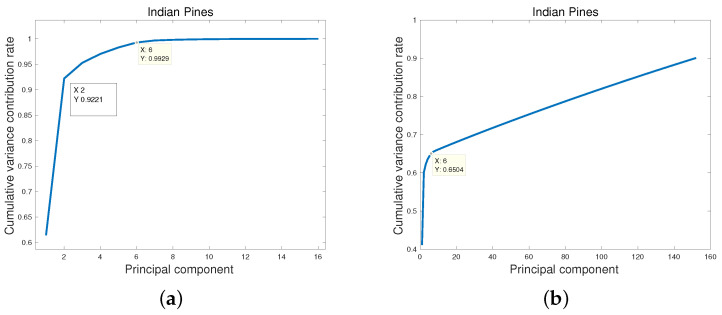
The cumulative variance contribution rate of principal components. (**a**) Clean Indian Pines; (**b**) Noisy Indian Pines.

In statistics, the CVCR of selected PCs must reach more than 0.8. However, this is impracticable in HSI processing. Figure 3 shows the CVCR of PCs of clean and noisy Indian Pines data. For a clean Indian Pines data, the CVCR of the first PC is 0.61, but the CVCR of the top two PCs is 0.92, which means top two PCs contain almost all information of Indian Pines data. For the noisy Indian Pines data, the CVCR of the first PC is 0.41 and the CVCR of top six PCs is 0.65, which suggests that more PCs are needed to explain the main information of Indian Pines data.

**Figure 4:**
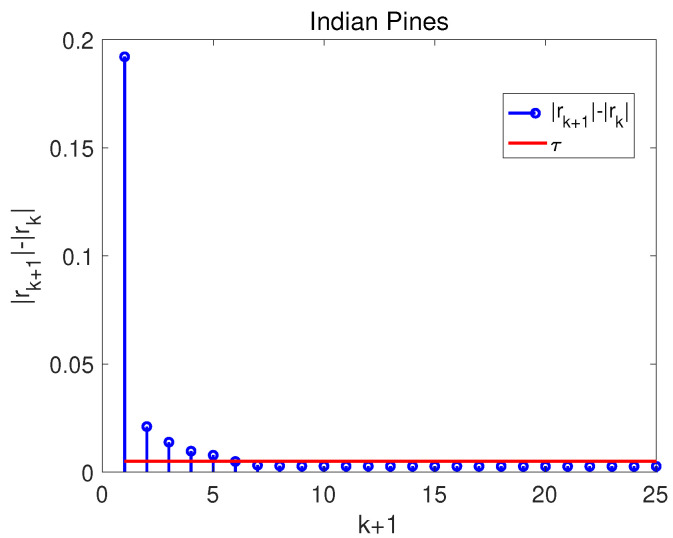
The “first small jump” scheme on noisy Indian Pines.

To solve the vital problem above, referring to “first significant jump” scheme [31,32], we propose a “first small jump” scheme to choose some PCs. The “first small jump” scheme looks for the smallest *k* such that

(3) $$|r_{k+1}\left|-\right|r_{k}|<\tau$$

where τ is a data-dependent value to detect the first small jump of the sequence $r_{k}$. The “jump” denotes the forward difference of the sequence $r_{k}$, in the left side of inequality (3). The scheme aims to find out the transition point of curve in Figure 3. According to the scheme (3), the selected top *k* PCs can explain the significant information of the HSI. For instance, in Figure 4 the value $|r_{7}\left|-\right|r_{6}|$ is firstly less than τ; then, top six PCs are selected. The selected top six PCs hold the larger CVCR than the first PC, and the other PCs that fail to provide useful information of the HSI are discarded.

The “first small jump” scheme is an adaptive method since the value τ is data-dependent rather than fixed. We simply set $\tau =p^{-1}{\left||r|\right|}_{1}$, where *p* is the band number of the HSI and $r=[r_{1},\ldots ,r_{k},\ldots ]$ is a vector whose elements are the CVCR of the PCs of the HSI. For different HSI data, τ is different.

Based on the selected PCs, the HSI is accurately divided along the spectral dimension by the superpixel segmentation method. The precise segmentation will make the obtained superpixel fibers have intensely low-rank properties. By considering the low-rank property of each superpixel fiber, we propose a HSI restoration model in the next section.

### 2.2. The HSI Restoration Model Based on Low-Rank Superpixel Fiber

The general HSI restoration model is formulated as follows:

(4) y=x+e+n,

where $y\in R^{m\times n\times p}$ and $x\in R^{m\times n\times p}$ are, respectively, denoted as the degraded and clean HSI; *e* stands for the mixture of strips, impulse noise, and dead lines; and *n* stands for the Gaussian noise.

After robust superpixel segmentation, the degraded HSI is divided into *K* non-overlapping superpixel fibers, as shown in Figure 1. Each superpixel fiber is an irregular tensor. Here, we unfold each superpixel fiber to a matrix for easy calculating. For the *i*th superpixel fiber, the restoration model is

(5) $$Y_{i}=X_{i}+E_{i}+N_{i},$$

where $Y_{i}$ and $X_{i}$ denote the two matrices, in which each column is the vectorized band of *y* and *x*, respectively. $E_{i}$ denotes the reshaped mixed noise. $N_{i}$ denotes the reshaped Gaussian noise. The clean *x* is finally obtained by merging all restored $X_{i}$. For convenience, the subscripts of the above formulation are temporarily omitted as follows:

(6) Y=X+E+N.

Each divided superpixel fiber contains the highly similar pixels and holds the high correlation across the spectra, so matrix X has intensely low-rank properties. Only some parts or bands of the HSI are contaminated by stripes, impulse noise, and dead lines; thus, matrix E is sparse [26]. The model (6) can be addressed by the following optimization problem:

(7) $$\min_{X,E}\;\;rank\left(X\right)+{\lambda ||E||}_{0},\;\;\;s.t.\;\;Y=X+E+N,$$

where λ is a regularized parameter. However, it is a NP-hard problem and cannot be solved directly.

There have been many articles that have confirmed that the weighted nuclear norm is a powerful tool in HSI restoration. We also adopt the weighted nuclear norm to explore the low-rank attributes of the superpixel fibers by displacing the rank function. Replacing the $\ell_{0}$ norm by the $\ell_{1}$ norm, the proposed model for superpixel fiber restoration is as follows:

(8) $$\min_{X,E}{\;\;||X||}_{w,*}+{\lambda ||E||}_{1},\;\;\;s.t.\;\;Y=X+E+N,$$

where

(9) $${\left||X|\right|}_{w,*}=\sum_{i}{\left|w_{i}\sigma_{i}\left(X\right)\right|}_{1}$$

is the weighted nuclear norm [33] of matrix X; $\sigma_{i}$ denotes the *i*th singular value of the matrix X, $w=[w_{1},\ldots ,w_{p}]$; and $w_{i}$ is the weight assigned to $\sigma_{i}\left(X\right)$.

Noteworthily, we adopt three types of weighting in our model.

- $w_{i}=\frac{c}{\sigma_{i}\left(X\right)+\varepsilon }$. $w_{i}$ is non-negative, where c>0 is a constant and $\varepsilon =10^{-16}$ is to avoid dividing by zero.
- w=[1,1,…,1]. The weighted nuclear norm is reduced to the standard nuclear norm, i.e., ${\left||X|\right|}_{w,*}={\left||X|\right|}_{*}$.
- w=[0,…,0,1,…,1]. The weighted nuclear norm becomes the partial sum of singular values, i.e.,
  $${\left||X|\right|}_{w,*}={\left||X|\right|}_{p=N}=\sum_{i=N+1}\sigma_{i}\left(X\right)$$
  where *N* is the target rank of matrix X [34].

In the proposed model (8), the weighted nuclear norm ${\left||X|\right|}_{w,*}$ characterizes the low-rank property of superpixel fiber and is a powerful tool to remove mixed noise. The $\ell_{1}$ norm of matrix E characterizes the sparse property of noise in the HSI. We will employ an iterative algorithm to alternately solve E and X. Finally, we match X back to the superpixel fiber and integrate all of the denoised superpixel fibers into the HSI *x*.

## 3. Optimization Algorithm

The ADM method [35] is very popular for solving convex or non-convex optimization models. The proposed model (8) is convex when w=[1,1,…,1]. However, it is non-convex when $w_{i}=\frac{c}{\sigma_{i}\left(X\right)+\varepsilon }$ and w=[0,…,0,1,…,1]. We use the ADM method to efficiently solve the proposed model, and the iterative optimization process is derived as follows.

The Lagrange function in the ADM frame is

(10) $$\begin{array}{c} L_{\mu }\left(X,E,Z\right)={\left||X|\right|}_{w,*}+{\lambda ||E||}_{1}+<Z,Y-X-E>+\frac{\mu }{2}||Y-X-{E||}_{F} \end{array}$$

where Z is the Lagrange multiplier and μ is a positive scalar. The minimization of the problem (8) can be optimized by alternately updating the variables E and X; the corresponding subproblems are as follows:

(11) $$\begin{array}{cc} X^{*} & =\arg\min_{X}L_{\mu }\left(X,E_{k},Z_{k}\right)\\ \; & =\arg\min_{X}\frac{1}{\mu }{\left||X|\right|}_{w,*}+\frac{1}{2}||X-\left(Y-E_{k}+\frac{1}{\mu }Z_{k}\right){\left|\right|}_{F}^{2}, \end{array}$$

(12) $$\begin{array}{cc} E^{*} & =\arg\min_{E}L_{\mu }\left(X_{k+1},E,Z_{k}\right)\\ \; & =\arg\min_{E}\frac{\lambda }{\mu }{\left||E|\right|}_{1}+\frac{1}{2}||E-\left(Y-X_{k+1}+\frac{1}{\mu }Z_{k}\right){\left|\right|}_{F}^{2}. \end{array}$$

For three types of weighting, the subproblem (11) can be solved by following three operators.

- For $w_{i}=\frac{c}{\sigma_{i}\left(X\right)+\varepsilon },i=1,\ldots ,p$,
  (13) $$X_{k+1}=D_{w_{k},\mu^{-1}}^{1}\left(Y-E_{k}+\mu^{-1}Z_{k}\right)$$
  where
  (14) $$D_{w_{k},\mu^{-1}}^{1}\left(L\right):=U{\left(\Sigma -\mu^{-1}\mathrm{diag}\left(w_{i}\right)\right)}_{+}V^{T}$$
  where $U\Sigma V^{T}$ is the singular value decomposition (SVD) of matrix L and ${\left(\alpha \right)}_{+}=\max\left\{\alpha ,0\right\}$. $D_{w_{k},\mu^{-1}}^{1}$ is the weighted singular value thresholding (WSVT) operator [33].
- For $w_{i}=1,i=1,\ldots ,p,$
  (15) $$X_{k+1}=D_{\mu^{-1}}^{2}\left(Y-E_{k}+\mu^{-1}Z_{k}\right)$$
  where
  (16) $$D_{\mu^{-1}}^{2}\left(L\right):=U{\left(\Sigma -\mu^{-1}I\right)}_{+}V^{T}$$
  where I is an identity matrix with the same size of Σ. $D_{\mu^{-1}}^{2}$ is the operator to solve standard nuclear norm.
- For $w_{i}=0,i=1,\ldots ,N,$ and $w_{i}=1,i=N+1,\ldots ,p,$
  (17) $$X_{k+1}=D_{N,\mu^{-1}}^{3}\left(Y-E_{k}+\mu^{-1}Z_{k}\right)$$
  where
  (18) $$D_{N,\mu^{-1}}^{3}\left(L\right):=U\left(\Sigma_{1}+{\left(\Sigma_{2}-\mu^{-1}I\right)}_{+}\right)V^{T}$$
  where
  $$\Sigma_{1}=\mathrm{diag}\left(\sigma_{1},\ldots ,\sigma_{N},0,\ldots ,0\right),$$
  $$\Sigma_{2}=\mathrm{diag}\left(0,\ldots ,0,\sigma_{N+1},\ldots ,\sigma_{p}\right).$$
  $D_{N,\mu^{-1}}^{3}$ is the partial singular value thresholding (PSVT) operator [34].

The subproblem (12) is convex and can be easily solved as

(19) $$E_{k+1}=S_{\lambda \mu^{-1}}\left(Y-X_{k+1}+\mu^{-1}Z_{k}\right)$$

where $S_{\lambda \mu^{-1}}\left(\alpha \right)=\mathrm{sign}\left(\alpha \right)\max\left(|\alpha |-\lambda \mu^{-1},0\right)$ is the soft-thresholding operator.

The whole solution process is summarized in Algorithm 1.
**Algorithm 1:** The HSI restoration algorithm based on low-rank superpixel fiber **Input:** the observed HSI *y*, the number of superpixel fibers *K*, the target rank *N*; **Output:** the restored HSI *x* and the sparse noise *e*; 1. Divide *y* by robust superpixel segmentation into *K* superpixel fibers and vectorize them as *K* matrices $Y^{1},\ldots ,Y^{K}$, like Figure 1; 2. **For** i=1:K do **Initialize**: $X_{0}^{i}=Y^{i}$, $E_{0}^{i}=0$, parameters λ>0, μ>0; 3. **Repeat**  $X_{k+1}^{i}=D_{w_{k},\mu^{-1}}^{1}\left(Y^{i}-E_{k}^{i}+\mu^{-1}Z_{k}^{i}\right)$;  or $X_{k+1}^{i}=D_{\mu^{-1}}^{2}\left(Y^{i}-E_{k}^{i}+\mu^{-1}Z_{k}^{i}\right)$;  or $X_{k+1}^{i}=D_{N,\mu^{-1}}^{3}\left(Y^{i}-E_{k}^{i}+\mu^{-1}Z_{k}^{i}\right)$;  $E_{k+1}^{i}=S_{\lambda \mu^{-1}}\left(Y^{i}-X_{k+1}^{i}+\mu^{-1}Z_{k}^{i}\right)$;  $Z_{k+1}^{i}=Z_{k}^{i}+\mu \left(Y-X_{k+1}^{i}-E_{k+1}^{i}\right)$;  **Until** convergence criterion is satisfied; 4. Return ${\hat{X}}^{i}=X_{k+1}^{i}$, ${\hat{E}}^{i}=E_{k+1}^{i}$;  **End** 5. Merge all ${\hat{X}}^{i}$ into the restored HSI *x*.


## 4. Experiments

To evaluate the performance of the proposed method, both simulated and real data experiments are conducted. The tensor dictionary learning (TDL) [36], LRTV [37], SSLRR [26], and E3DTV [38] methods are utilized to compare with the proposed approach. All parameters involved in the competing methods are chosen as described in the reference papers. In the proposed approach, we set $\lambda =\frac{1}{\sqrt{max(p,q)}}$ and $\mu =(\sqrt{p}+\sqrt{q})\delta$, where *p* and *q* denote the column number and row number of the matrix X, and δ is the deviation of Gaussian noise. Due to the shape of each superpixel fiber is different, the value of *q* is different. Algorithm 1 typically takes far less iterations to satisfy the condition $\frac{\left|\right|X_{k}-X_{k-1}{\left|\right|}_{F}^{2}}{X_{k}}>\varepsilon$.

The proposed method based on the standard nuclear norm (solved by $D_{\mu^{-1}}^{2}$ operator) is unconsidered in experiments. As is known, its denoising performance is poorer than the one of the weighted nuclear norm. The ERS method is applied in robust superpixel segmentation to divide the noisy HSI in following experiments. The usual image quality metrics MPSNR, MSSIM, ERGAS [39], and runtime are adopted to evaluate the restoration results. The bigger MPSNR and MSSIM values, the better the denoising effect. The smaller the ERGAS value, the closer the restored HSI is to the original HSI.

### 4.1. Simulation Experiment

The public hyperspectral and multispectral image data are used to demonstrate the performance of the proposed method, i.e., Indian Pines (145 × 145 pixels and 224 spectral bands) and pompoms (512 × 512 pixels and 31 spectral bands). The same percentage of salt-and-pepper impulse noise and the same distribution of zero-mean Gaussian noise is added into each band. In addition, dead lines are added into some bands, as in the following two cases.

Case 1: (Gaussian Noise + Impulse Noise) Both Gaussian noise with the variance of 0.1 and impulse noise with the percentage of 0.1 are added to the Indian Pines data.
Case 2: (Gaussian Noise + Impulse Noise + Dead Lines) Both Gaussian noise with the variance of 0.1, impulse noise with the percentage of 0.1, and dead lines from band 10 to band 25 are added into pompoms data.

**Figure 5:**
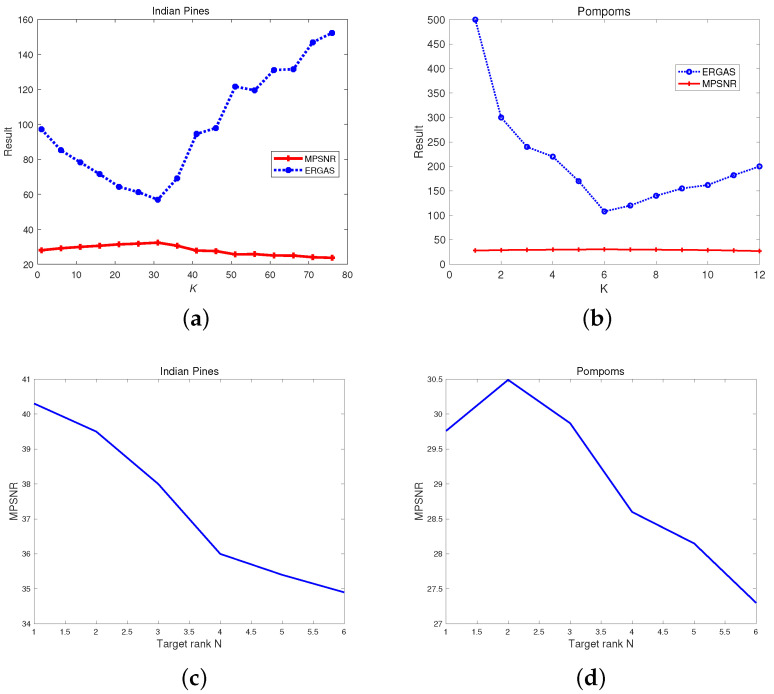
Relationship of superpixel fibers’ number *K*, target rank *N*, ERGAS, and MPSNR. (**a**) *K* vs. MPSNR and ERGAS on Indian Pines, (**b**) *K* vs. MPSNR and ERGAS on pompoms, (**c**) *N* vs. MPSNR on Indian Pines, and (**d**) *N* vs. MPSNR on pompoms.

In simulated experiments, the number of superpixel fibers *K* and the target rank *N* are vital parameters in the proposed method. For two cases above, Figure 5 shows the relationship between MPSNR, the ERGAS values, and the superpixel fibers’ number *K*, and the relationship between the MPSNR value and the target rank *N*. We can see that when K=34 and N=1, the MPSNR values are biggest, and the ERGAS value is the smallest, i.e., the proposed method achieves the best results for Indian Pines data. Thus, we set K=34 in the proposed method based on the WSVT operator, and K=34 and N=1 in the proposed method based on PSSV operator. In the same way, K=16 and N=2 are set in the proposed method for restoring pompoms data.

Noteworthily, the ERGAS value is very large when K=1 (no superpixel segmentation). This means that only considering low-rank prior without superpixel segmentation will not adequately restore the HSI. With increasing *K*, the MPSNR value gradually becomes big and the ERGAS value becomes small, which suggests that robust superpixel segmentation works, and combined with the weighted nuclear norm it can obtain better HSI restoration results. However, too many superpixel fibers (large *K*) may lead to biased segmentation. Thus, when *K* is bigger, the ERGAS value starts to grow, i.e., the denoising performance starts to decline.

**Figure 6:**
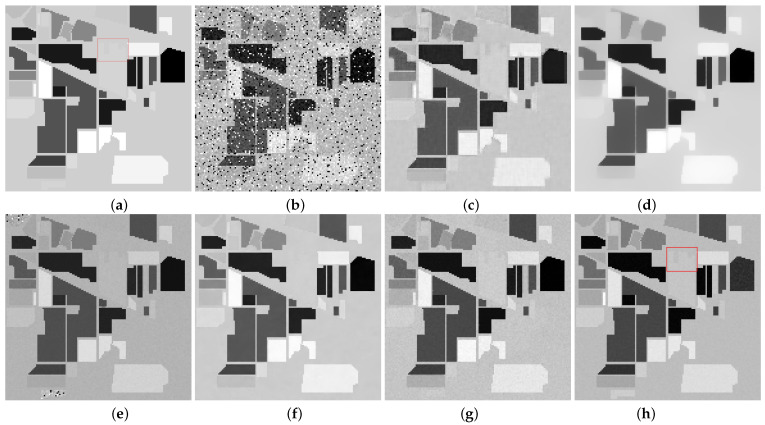
Restoration results on Indian Pines. (**a**) Original band 95, (**b**) noisy band 95, (**c**) TDL, (**d**) LRTV, (**e**) SSLRR, (**f**) E3DTV, (**g**) proposed (WSVT), and (**h**) proposed (PSVT).

**Figure 7:**
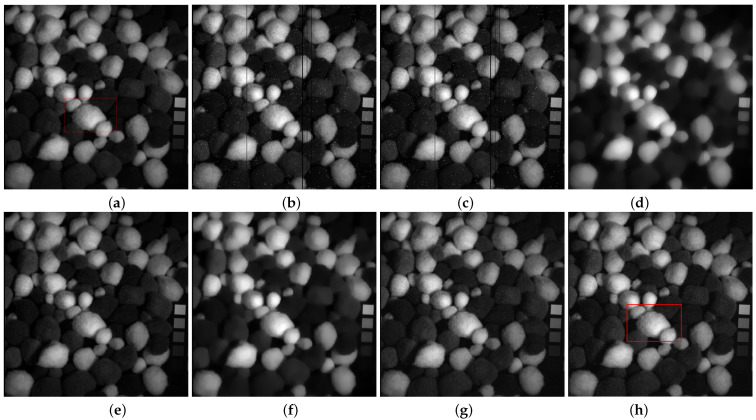
Restoration results on pompoms. (**a**) Original band 23, (**b**) noisy band 23, (**c**) TDL, (**d**) LRTV, (**e**) SSLRR, (**f**) E3DTV, (**g**) proposed (WSVT), and (**h**) proposed (PSVT).

**Figure 8:**
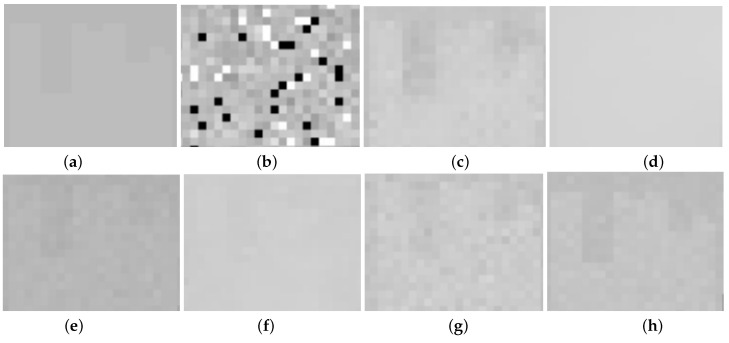
Enlargement of the red box in Figure 6. (**a**) Original band 95, (**b**) noisy band 95, (**c**) TDL, (**d**) LRTV, (**e**) SSLRR, (**f**) E3DTV, (**g**) proposed (WSVT), and (**h**) proposed (PSVT).

**Figure 9:**
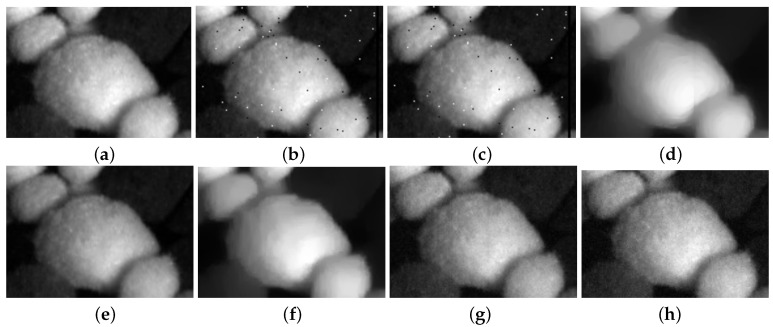
Enlargement of the red box in Figure 7. (**a**) Original band 23, (**b**) noisy band 23, (**c**) TDL, (**d**) LRTV, (**e**) SSLRR, (**f**) E3DTV, (**g**) proposed (WSVT), and (**h**) proposed (PSVT).

Figure 6 and Figure 7 present the restoration results of all test methods on Indian Pines data and pompoms data. Figure 8 and Figure 9, respectively, display the enlarged images of the red box in Figure 6 and Figure 7. In Figure 6, all compared methods can remove a significant amount of mixed noise. However, some boundaries in the restored band obtained by TDL are blurred, and some edges in the restored band obtained by LRTV are lost. The restored band of SSLRR still contains a litter noise. The restored band of the proposed approach based on WSVT operator is not smooth. In Figure 8, while E3DTV removes almost all of the noise, the proposed approach based on the PSVT operator retains more details and edges, and its restored band is closer to the original band.

In Figure 7, TDL fails to remove mixed noise. The restored band obtained by LRTV is blurred. E3DTV wipes off the mixed noise well but also smooth some edges. Visually, the restored bands achieved by the proposed method are clearer than the one of SSLRR, which confirms that the robust superpixel segmentation is better for HSI restoration than superpixel segmentation just using the first PC. In Figure 9, we can see that the proposed method not only removes the mixed noise but also recovers the vivid images.

The statistical data reporting the performance of all test methods are shown in Table 1 and Table 2. The highest MPSNR value and MSSIM value, the lowest ERGAS value, and the least time are highlighted in bold. From two tables, we can see that the proposed method based on the PSVT operator with the highest MPSNR and MSSIM values outperforms other methods, which is consistent with the Figure 6 and Figure 7. This demonstrates that robust superpixel segmentation combined with the weighted nuclear norm achieves the best performance on mixed-noise removal.

**Figure 10:**
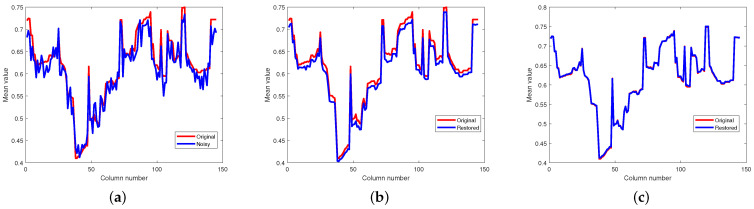
Comparison results on horizontal mean profiles of Indian Pines. (**a**) Noisy, (**b**) proposed (WSVT), and (**c**) proposed (PSVT).

**Figure 11:**
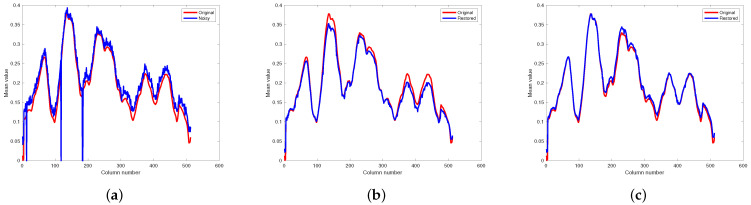
Comparison results on horizontal mean profiles of pompoms. (**a**) Noisy, (**b**) proposed (WSVT), and (**c**) proposed (PSVT).

Figure 10 and Figure 11, respectively, display the horizontal mean profiles of Indian Pines data in case 1 and pompoms data in case 2. The horizontal axis and the vertical axis refer to the column number of the HSI and the mean values of each column, respectively. The red curve stands for the value of the original and clean HSI, and the blue curve stands for the value of the restored HSI. We observe that both for Indian Pines data and pompoms data, the proposed method based on the PSVT operator presents a smooth curve close to the original one, meaning that the mixed noise can be effectively suppressed.

**Figure 12:**
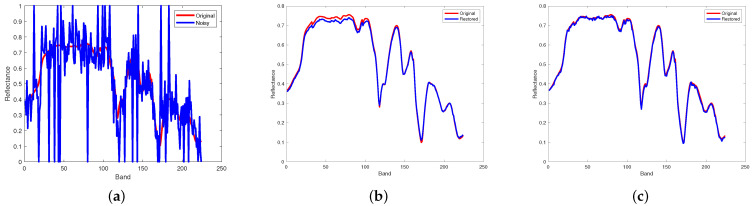
Comparison results on spectral signature of pixel (10, 10) in Indian Pines. (**a**) Noisy, (**b**) proposed (WSVT), and (**c**) proposed (PSVT).

**Figure 13:**
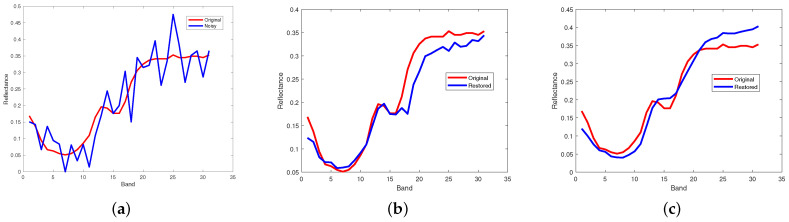
Comparison results on spectral signature of pixel (100, 100) in pompoms. (**a**) Noisy, (**b**) proposed (WSVT), and (**c**) proposed (PSVT).

Figure 12 and Figure 13 respectively illustrate the spectral signature of pixel (10, 10) in Indian Pines data and pixel (100, 100) in pompoms data. The rapid fluctuations of the blue curve indicate the existence of mixed noise. In Figure 12c, it can be clearly observed that the blue curve is very matched by the red one, which means that the restored pixel by the proposed method based on the PSVT operator is nearly the same as the pixel of the original Indian Pines data. Unfortunately, not all pixels can be recovered precisely by the proposed method, such as the pixel (100, 100) in the pompoms data.

### 4.2. Real Data Experiment

Urban data and Botswanna data are popular in the HSI denoising experiments [40,41]. We also use them in real data experiments. Only SSLRR and E3DTV are employed as comparison methods, due to the performances of LRTV and TDL being poor in simulated experiments. In the proposed method, we set K=12, N=2 for urban data and K=4, N=2 for Botswanna data.

**Figure 14:**
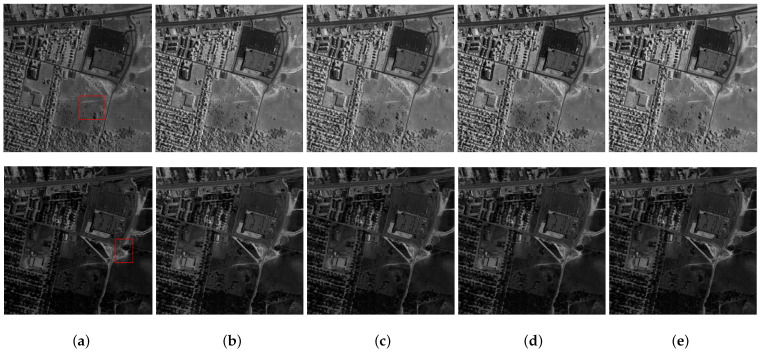
Restoration results of band 87 and band 156 on urban. (**a**) Noisy, (**b**) SSLRR, (**c**) E3DTV, (**d**) proposed (WSVT), and (**e**) proposed (PSVT).

Urban data has 307 × 307 pixels and 210 spectral bands ranging from 400 nm to 2500 nm. It contains strong noise in some bands, such as deadlines, stripes, Gaussian noise, and other unknown noise types. Figure 14a shows the band 87 and band 156 of the urban data. We observed that band 87 is contaminated by several horizontal lines, and band 156 is corrupted by stripes and Gaussian-like random noise.

**Figure 15:**
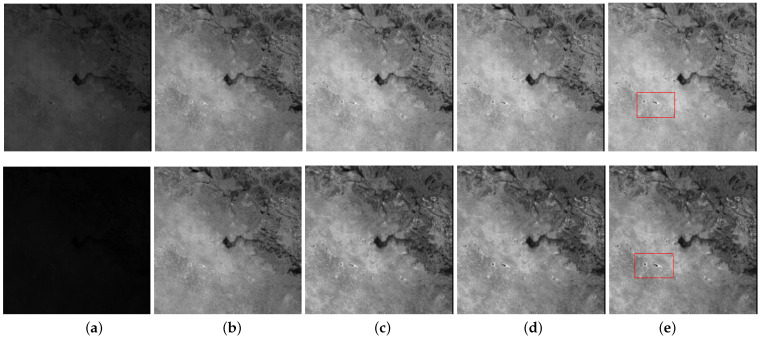
Restoration results of band 68 and band 110 on Botswana. (**a**) Noisy, (**b**) SSLRR, (**c**) E3DTV, (**d**) proposed (WSVT), and (**e**) proposed (PSVT).

Botswanna data was collected in 2001 across Okavango Delta, Botswana (BOT) and has a size of 1476 × 256 pixels and 242 spectral bands ranging from 357 to 2576 nm [34]. We use a subimage of size 256 × 256 × 145, where some noisy and water absorption bands are discarded. Figure 15a illustrates the band 68 and band 110 of Botswanna data. We observed that band 68 and band 110 are heavily corrupted by water absorption and unknown noise, which make the denoising task more challenging.

**Figure 16:**
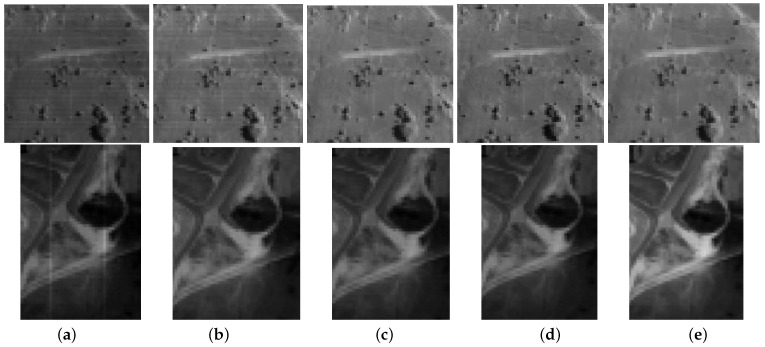
Enlargement of the red box in Figure 14. (**a**) Noisy, (**b**) SSLRR, (**c**) E3DTV, (**d**) proposed (WSVT), and (**e**) proposed (PSVT).

Figure 14 shows the restoration results of four HSI denoising methods on urban data. Figure 16 presents the enlarged images of the red box in Figure 14. We observe that SSLRR fails to remove the horizontal lines in band 87 although it suppresses most of the noise in Figure 16. E3DTV discards some details and makes some edges smooth in restored bands 87 and 156. The proposed strategy presents remarkable ability regarding mixed-noise removal and restores the clear and vivid bands.

In Figure 15, restored bands 68 and bands 110 obtained by four HSI denoising methods on Botswana data are displayed. SSLRR suppresses some of the noise but cannot remove it completely. E3DTV works well for noise removal, but the detailed spatial information is lost. The proposed approach efficiently removes the unknown noise and better preserves the details, as shown in the red box in Figure 15e.

## 5. Discussion

**Figure 17:**
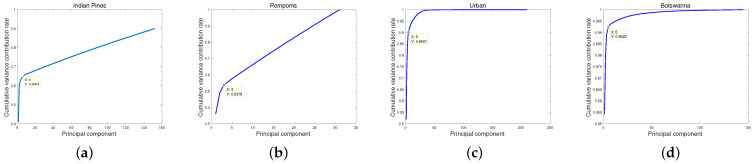
The cumulative variance contribution rate of principal components. (**a**) Noisy Indian Pines, (**b**) noisy pompoms, (**c**) urban, and (**d**) Botswanna.

There are four test data in our experiments, i.e., noisy Indian Pines, noisy pompoms, and urban and Botswanna data. Figure 17 shows the CVCR of PCs of four test data. According to the “first small jump” scheme, we, respectively, select top five PCs for noisy Indian Pines data and top three PCs for noisy pompoms data in simulated experiments, the top five PCs for the urban data, and the top five PCs for the Botswanna data in real data experiments. It is suggested that value τ in “first small jump” scheme is data-dependent rather than fixed.

In particular, in Figure 17c, the CVCR of the top five PCs is 0.9007, which means that the top five PCs can fully explain the urban data. Likewise, as shown in Figure 17d, the CVCR of the top five PCs is 0.9922, and they also fully explain the Botswanna data. It is suggested that the “first small jump” scheme is reasonable and feasible, especially for real HSI data.

For the proposed approach based on PSVT operator, Table 3 reports the relationship between the number of PC and MPSNR values when recovering the noisy Indian Pines and pompoms data, respectively. Obviously, when just selecting the first PC, the MPSNR values are low for two noisy data. When selecting top five PCs for noisy Indian Pines data and top three PCs for noisy pompoms data, the proposed method performs best with the highest MPSNR value. This demonstrates that robust superpixel segmentation combined with the weighted nuclear norm obtained the best performance.

**Figure 18:**
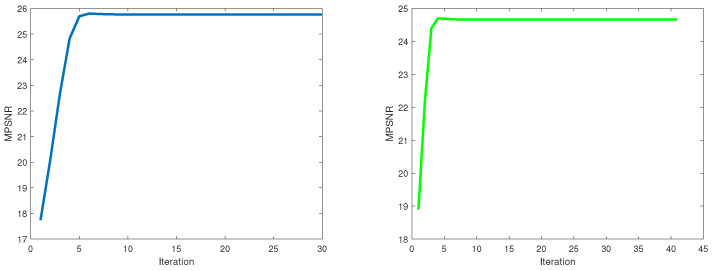
Iteration numbers vs. MPSNR for any two superpixel fibers restoration.

Since the weighted nuclear norm is non-convex, the convergence of the proposed method is hard to proof in theory. However, the proposed method based on the WSVT operator can obtain a fixed-point solution if the weights *w* are in a non-descending order [42]. Empirically, the proposed method based on the PSVT operator is convergent when restoring each superpixel fiber. Figure 18 shows the relationship of the iteration numbers and the MPSNR value obtained by the proposed method based on the PSVT operator for any two superpixel fibers’ restoration. It can be seen that the proposed method based on the PSVT operator is fast convergent.

## 6. Conclusions

Since the accurate superpixel segmentation result will enhance the low-rank property of the HSI, in this paper, we have proposed a robust superpixel segmentation strategy using the “first small jump” scheme to more precisely divide the HSI. Then, the weighted nuclear norm is employed to characterize the low-rank property of the superpixel fibers. We have summarized the weighted nuclear norm by three types of weighting, and adopted the WSVT operator, PSVT operator, and soft-thresholding operator to solve the proposed HSI restoration model in the ADM frame. The extensive experiments on simulated and real HSIs demonstrate that our approach performed best, in both visual and quantitative assessments.

## Figures and Tables

**Table 1:** Experimental results (MPSNR(dB), MSSIM, ERGAS, and time(s)) on noisy Indian Pines.

Method	MPSNR	MSSIM	ERGAS	Time
TDL	28.71	0.925	87.59	**6.58**
LRTV	26.51	0.908	113.05	81.16
SSLRR	30.48	0.960	70.48	11.37
E3DTV	28.84	0.978	91.67	46.58
proposed (WSVT)	31.50	0.980	46.26	19.12
proposed (PSVT)	**32.27**	**0.996**	**30.10**	24.87

**Table 2:** Experimental results (MPSNR(dB), MSSIM, ERGAS, and time(s)) on noisy pompoms.

Method	MPSNR	MSSIM	ERGAS	Time
TDL	24.39	0.737	220.45	185.22
LRTV	24.17	0.735	224.89	351.51
SSLRR	24.93	0.651	208.47	**8.39**
E3DTV	29.93	0.904	144.23	55.93
proposed (WSVT)	28.84	0.909	149.54	63.58
proposed (PSVT)	**30.47**	**0.948**	**108.10**	32.00

**Table 3:** Experimental results on noisy Indian Pines and pompoms.

Noisy Indian Pines		Noisy Pompoms	
Number of PC	MPSNR	Number of PC	MPSNR
1	30.41	1	24.41
3	31.39	2	30.24
**5**	**32.27**	**3**	**30.47**
6	32.24	4	30.41
7	32.01	5	30.39

## Data Availability

The HSI data used in our experiments are freely available from http://www.ehu.eus/ccwintco/index.php/Hyperspectral_Remote_Sensing_Scenes (accessed on 27 June 2012).
